# Evolutionary Kuramoto dynamics

**DOI:** 10.1098/rspb.2022.0999

**Published:** 2022-11-09

**Authors:** Elizabeth A. Tripp, Feng Fu, Scott D. Pauls

**Affiliations:** ^1^ Department of Mathematics, Sacred Heart University, Fairfield, CT 06825, USA; ^2^ Department of Mathematics, Dartmouth College, Hanover, NH 03755, USA; ^3^ Department of Biomedical Data Science, Geisel School of Medicine at Dartmouth, Lebanon, NH 03756, USA

**Keywords:** evolutionary game theory, synchronization, oscillatory dynamics

## Abstract

Biological systems have a variety of time-keeping mechanisms ranging from molecular clocks within cells to a complex interconnected unit across an entire organism. The suprachiasmatic nucleus, comprising interconnected oscillatory neurons, serves as a master-clock in mammals. The ubiquity of such systems indicates an evolutionary benefit that outweighs the cost of establishing and maintaining them, but little is known about the process of evolutionary development. To begin to address this shortfall, we introduce and analyse a new evolutionary game theoretic framework modelling the behaviour and evolution of systems of coupled oscillators. Each oscillator is characterized by a pair of dynamic behavioural dimensions, a phase and a communication strategy, along which evolution occurs. We measure success of mutations by comparing the benefit of synchronization balanced against the cost of connections between the oscillators. Despite the simple set-up, this model exhibits non-trivial behaviours mimicking several different classical games—the Prisoner’s Dilemma, snowdrift games, coordination games—as the landscape of the oscillators changes over time. Across many situations, we find a surprisingly simple characterization of synchronization through connectivity and communication: if the benefit of synchronization is greater than twice the cost, the system will evolve towards complete communication and phase synchronization.

## 1. Introduction

Biological rhythms are ubiquitous, providing timing mechanisms that guide sequential processes [1]. In many cases, these take the form of clocks that are synchronized with external environmental cues to provide a *circadian* signal—one that allows the organism to predict the local daily light/dark cycle. Such a coherent circadian signal confers many advantages—mammals can anticipate changes in light that allow them to avoid predators, find food and generally increase their chances of survival. This basic principle informs natural selection: the existence of a variety of mechanisms to generate circadian signals—ranging from simple intracellular mechanisms in single-cell organisms to more complex multicellular or multi-organ systems in insects, birds, mammals and other species—demonstrates that the evolutionary benefit of such a system often outweighs its cost. On the other hand, for some organisms, like the eyeless Mexican cavefish, possessing a circadian clock would not confer the same kind of benefit, and thus such a system is not present [2].

We focus on the mammalian circadian system, which is governed by a central ‘master clock’ called the suprachiasmatic nucleus (SCN), a small centre in the brain that sits just above the optic chiasm. It receives light/dark signals from the optic nerve and uses them to generate and maintain the organism’s circadian rhythm. Most of the roughly 20 000 neurons in the SCN are oscillatory, exhibiting approximately 24 h rhythms (see [3] for a review of the basic neurobiology). When their connectivity is disrupted, the neurons oscillate with about the same period but randomly out of phase. However, when connectivity is intact, the oscillations exhibit phase-locked synchronization [4]. The nature and function of the interconnectivity between the neurons is of fundamental importance in the study of the SCN, and we develop tools and generate initial results in assessing the role that evolution plays in generating and constraining the structure of the SCN.

Mathematically, we view the mammalian SCN as a network of coupled oscillators (used interchangeably with oscillatory neurons hereafter). Understanding the synchronization of systems of coupled oscillators has a rich history in the study of dynamical systems and applications in numerous fields [5,6]. Decades of work has demonstrated the interplay between the properties of these systems and their ability to synchronize (surveys of the field include [7–9]). One of the simplest and most fruitful modelling approaches uses a differential equations system model first introduced by Kuramoto [10]. While an elegant analytic approach exists in the case of two oscillators, with more oscillators and more complicated connectivity, analysis of the Kuramoto system becomes (much) harder. While we can understand this system (and some variants) analytically when the coupling topologies are particularly simple [8] and/or when we look at the mean-field limit as the number of oscillators tends to infinity [7,8], more complex (and biologically plausible) connectivity patterns are not tractable. For these types of cases, we rely on numerical approximation of solutions which can be both difficult and costly computationally.

We instead approach the problem using techniques from the field of evolutionary game theory (EGT), which applies classical game theory to the study of evolving populations [11–13]. The competitive advantages of various traits (or *strategies*, in the language of EGT) that exist in the population are based on payoffs accrued from pairwise or multi-person game interactions between connected individuals. Traits of individuals are allowed to evolve over time, mimicking the biological process of natural selection, and so traits that confer a competitive advantage have greater success at propagating as the population evolves. This framework is designed to answer questions about which population traits are more evolutionarily successful, and under what conditions particular traits are advantageous. When we view the neurons of the SCN as our population of interest, this EGT set-up lends itself naturally to our main question: what conditions allow for the synchronization behaviour observed in the mammalian SCN to arise? While the benefit conferred to the organism from this synchronization is shared by each neuron, the communication between neurons that enables synchronization behaviour is costly. Thus, each neuron faces a trade-off that the evolutionary process can resolve. This kind of trade-off is captured in classic cooperative dilemmas, such as Prisoner’s Dilemma game [13,14].

Antonioni & Cardillo [15] incorporate EGT into the Kuramoto framework using a modified version of Prisoner’s Dilemma. In their model, neurons have two possible strategies: cooperation, where neurons influence each other to move towards synchronization, and defection, where they do not. They use solutions of Kuramoto coupled oscillator systems to calculate payoffs of a neuron’s strategy based on the level of local synchronization. In contrast to the typical EGT set-up, where an individual’s payoff is dependent on the current state of the system and the strategy of their opponent, in this framework, a neuron will receive the same payoff regardless of the strategy of its particular opponent as the payoff function depends on the strategies and phases of all of its neighbours.

While Antonioni and Cardillo’s framework captures the behaviour of the oscillating neurons as they work to synchronize given the tension inherent to such a system, it is not set up to answer evolutionary questions. The fixed descriptions of the payoff parameters [15,16], which are restricted to the type of a Prisoner’s Dilemma game, do not allow for the study of the explicit payoff conditions under which synchronization is most likely to occur. As the payoff is determined by numerical solutions to Kuramoto systems, it is intractable to derive closed-form conditions for natural selection to favour synchronization across a wide variety of scenarios.

To address this issue, we allow the payoff parameters a range of possible values, subject to a few biologically plausible assumptions (see details in Methods and Models). This allows us to work backwards and discover what values of the payoff parameters cause the system to evolve into a state of synchronization, which in turn allows us to make inferences about the biology of the mammalian SCN. Additionally, while the framework of [15] allows for the synchronization process to occur separately from the evolutionary dynamics of the neurons’ strategies, the computational cost of accurately solving Kuramoto systems over long time frames is high for large populations of oscillators. In light of this, we consider intrinsic phases of neurons and their communicative strategies as combined traits that jointly determine their payoffs and are subject to natural selection. This novel set-up leads to coevolutionary dynamics of communicative strategies and multiple discrete phases of neurons, thereby providing a framework for a new model of coupled oscillatory systems that allows us to study the impact of the evolutionary constraint on the population of oscillators. Consequently, we will be able to determine what evolutionary constraints allow organisms to develop this synchronized oscillatory behaviour.

In this paper, we study the simplest case where oscillators either communicate with all other oscillators or none. We define a game, also inspired by Prisoner’s Dilemma [17], in which each oscillator receives a payoff based on their current level of synchrony with their neighbouring oscillators and whether or not they choose to communicate to improve the synchrony of the region. Using techniques from evolutionary games in finite populations [18–21] coupled with simulation, we are able to determine when communication is a favourable strategy for the population. We find that, under a variety of assumptions, this choice to communicate—and thus synchronize—is favoured when the benefit received by two synchronized, communicating neurons exceeds twice the neuron’s incurred cost of communication. Our analysis also sheds light on the possible connectivity structures, as measured by the communication profiles of the oscillators, that are consistent with the evolutionary constraints. We find that complete communication, complete non-communication, and a mixture of both are viable outcomes under a range of biologically plausible assumptions.

## 2. Methods and models

In thinking about the possible structures for the SCN, we use a common coupled oscillator model due to Kuramoto [10] as a starting point. We define *n* oscillators that are characterized by their intrinsic frequencies, {*ν*_1_, …, *ν*_*n*_}, and output oscillatory phases, {*ϕ*_1_, …, *ϕ*_*n*_}. These oscillators are coupled together with different strengths, where the impact of oscillator *k* on oscillator *j* is recorded as *h*_*jk*_.

In light of the tendency towards synchronization in a suitably constructed Kuramoto model, we propose a new approach to understanding synchronization of coupled oscillatory systems using EGT. The competitive advantages of various traits that exist in the population are encoded as a pairwise game between connected individuals. Traits of individuals are allowed to evolve over time, mimicking the biological process of natural selection. Traits that confer an individual with a competitive advantage have greater success at propagating as the population evolves.

### (a) Oscillator traits

How do we translate the ideas behind the Kuramoto model into the EGT framework? We have two goals. The first is to preserve features of the Kuramoto dynamics when building the evolutionary dynamics. The second is to allow the evolutionary mechanism to change the connectivity over time, reflecting the competitive advantage of synchronous oscillation. The Kuramoto model has two sets of parameters: the {*ν*_*i*_} and the *h*_*ij*_. We make two simplifying assumptions: that the frequencies are identical and that the connection strengths are either one or zero. We associate two traits with each oscillator—a phase and a measure of communicability. The phase trait encodes the analogue of the Kuramoto phase evolution but within evolutionary dynamics while the communicativity gives a mechanism to turn connectivity between oscillators on and off.

### (b) Strategies and payoffs

Trait evolution is achieved through setting up different benefits and costs for a game that oscillators play with their neighbours. We rely on two principles

— Oscillators benefit from synchronization with their neighbours: the closer the phases of the two oscillators, the greater the benefit;
— For one oscillator to influence another, the influencing oscillator must be communicative, which incurs a cost.

These two principles are in tension: communicative neurons can align their phases with their neighbours raising their benefit but incur a cost of doing so, while non-communicative oscillators can reap the benefit of aligned phases without incurring the cost. This type of tension is present in many of the classical game theoretic analyses—for example, Prisoner’s Dilemma—and creates the opportunity for different outcomes depending on the parameters of the model.

To encode these principles, we define a game on *n* oscillators. The strategy for oscillator *i* is *S*_*i*_ = (*s*_*i*_, *ϕ*_*i*_), a binary communication strategy where *s*_*i*_ ∈ {*N*, *C*} and $\phi_{i}\in \{j(2\pi /d){\}}_{\;j=1}^{d}$ is the associated phase. To describe the payoffs, we construct a 2*d* × 2*d* payoff matrix containing the payoffs of the different strategic pairings. Oscillator *j*’s payoff depends on its communication strategy, the communication strategy of its partner, *k*, and the cyclic difference in their phases, denoted Δ*ϕ*_*jk*_.^1^ A restricted payoff matrix describing pairwise interactions between oscillators can take on one of the three following possible forms:

2.1 $$\begin{array}{rl} & \begin{array}{ccc} \left(I\right) & \left(C,\phi_{j}\right) & \left(C,\phi_{k}\right)\\ \left(C,\phi_{j}\right) & B(0)-c & B(\Delta \phi_{\;jk})-c\\ \left(C,\phi_{k}\right) & B(\Delta \phi_{\;jk})-c & B(0)-c \end{array},\\ & \begin{array}{ccc} \left(\mathrm{II}\right) & \left(C,\phi_{j}\right) & \left(N,\phi_{k}\right)\\ \left(C,\phi_{j}\right) & B(0)-c & \beta (\Delta \phi_{\;jk})-c\\ \left(N,\phi_{k}\right) & \beta (\Delta \phi_{\;jk}) & 0 \end{array},\\ \mathrm{and}\; & \begin{array}{ccc} \left(\mathrm{III}\right) & \left(N,\phi_{j}\right) & \left(N,\phi_{k}\right)\\ \left(N,\phi_{j}\right) & 0 & 0\\ \left(N,\phi_{k}\right) & 0 & 0 \end{array} \end{array}\}$$

Here, *c* represents the cost of communication and the functions *B* and *β* encode the benefit accrued by oscillators. We assume both functions are monotone decreasing to reflect the assumption that the benefit is larger when phases are similar and distinguish between the benefit gained when two communicative oscillators are interacting (using the function *B*) and when only one of them communicates (using *β*). While it is often reasonable to assume that *B* and *β* are identical, more generally we assume only that *B*(Δ*ϕ*) ≥ *β*(Δ*ϕ*). The full payoff matrix is composed of four *d* × *d* blocks, where the entries in the top left *d* × *d* block are given by the matrix (I) and those in the top *d* × *d* off-diagonal block and those in the bottom left block from the matrix (II), while the final block contains the zero matrix (i.e. matrix (III)).

To simplify the description of the matrix, we observe that angular differences of phases are both symmetric and exhibit a cyclic symmetry as well. We write B,β : {0,1,2,…}→R where the natural numbers in the domain represent multiples of the angular distance between two phases: we let *B*(Δ*ϕ*_qr_) = *B*(*k*) (or *β*(Δ*ϕ*_qr_) = *β*(*k*)), where *k* = |*q* − *r*| if |q−r|≤⌊(d/2)⌋, otherwise, if |q−r|>⌊(d/2)⌋, *k* = *d* − |*q* − *r*| . The matrix in the electronic supplementary material, §2 shows the entire payoff matrix with these notational conventions.

### (c) Evolution of the system

Changes to the strategies are governed by a Moran process (also known as the birth–death process): at each time step, we choose one oscillator with probability proportional to its fitness to reproduce, and select another oscillator (with replacement that includes the parent) uniformly at random to be replaced by the newly produced offspring [13]. This evolutionary process allows both the phase and communicativity to change simultaneously, which we view as a model of social learning. Allowing the phase to change via this process provides the system the opportunity to move towards synchronization (or not) while changes in the communicativity are changes to the connectivity of the network.

Here again, we make simplifying assumptions to facilitate the analysis of the system. First, by construction, these two traits evolve simultaneously on the same timescale. This is not entirely biologically plausible as phases change within short time frames (e.g. hours or days) while the communicativity changes on the typical evolutionary timescale of generations. While this limits the interpretability of the results biologically, it allows for a deep and precise description of the outcomes of the system that provide a baseline for further investigation.

While the payoff matrices in (2.1) above allow for pairwise comparisons of the relative strengths of various strategies, other quantities allow for more overarching comparisons. The *expected payoff* of a strategy *E*, denoted by *π*_*E*_, calculates the average payoff received by strategy *E* given the current frequency of each strategy among its neighbours. The *fitness* of an oscillator with strategy *E* is given by $f_{E}=e^{\delta \pi_{E}}$, where the parameter *δ* is the strength of selection [21]. The fitness of an oscillator’s strategy is used to weigh the oscillator’s ability to reproduce its strategy in the evolution of the population, thereby mimicking the effect of natural selection on advantageous traits in biological evolution.

We allow the strategy of a new oscillator the chance to mutate during each reproduction step, meaning that with small probability *μ*, the new oscillator will be assigned a random strategy rather than faithfully inherit the strategy of its parent. The strength of selection, *δ*, determines the extent to which the structure of the game impacts the evolutionary success of each strategy: large values of *δ* give more weight to the role of payoff in fitness, while a selection strength of *δ* = 0 gives all individuals the baseline fitness value of 1. The latter process is called *neutral drift* [13], since the game plays no role in an individual’s reproductive success. Thus, any oscillator with a strategy with high average payoff (and thus higher fitness) will be more likely to reproduce at each time step in the evolutionary process. Over time, we expect strategies with higher fitness values to increase in number in the population. Eventually, the Moran process will reach equilibrium in a state where all neurons share the same strategy [13]. Once reaching such an equilibrium point, the state of the system will remain unchanged, unless a mutation event occurs.

As communication should promote synchronization, we look for those conditions under which (C,⋆) strategies are selectively favoured. In this work, we assume the simplest possible underlying network topology (the so-called well-mixed population [18]): all oscillators are equally likely to interact with all other oscillators in the population.

### (d) Methods of analysis

**Figure 1.**
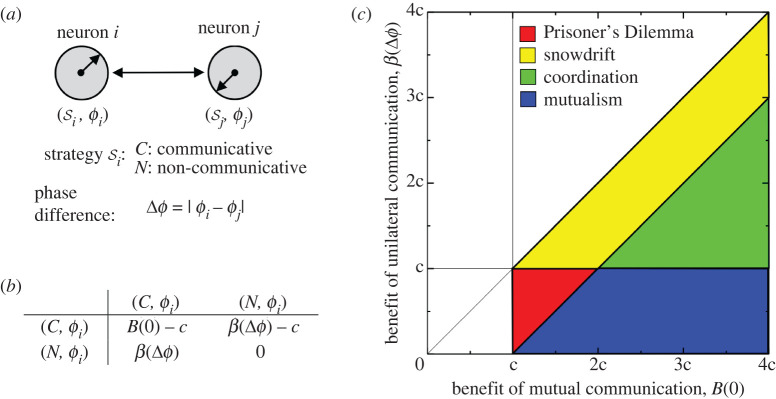
We study the coevolution of communicative strategies of oscillators and their phase synchronization through the lens of evolutionary game theory. (*a*) The pairwise interactions between any two oscillators that are modulated by their communicative strategies and phases, (*s*_*i*_, *ϕ*_*i*_). We consider binary communicative strategies, namely, *s*_*i*_ = *C* if *i* is committed to communication at a cost *c*, and *s*_*i*_ = *N* if *i* is non-communicative and pays no cost. The benefit each received from their interactions depends on both their communicative strategies and phase difference Δ*ϕ*_*ij*_. If both *i* and *j* are communicative neurons, the benefit they each will receive is *B*(Δ*ϕ*_*ij*_) = *B*(0)[1 + cos (*ϕ*_*i*_ − *ϕ*_*j*_)]/2. If only one of *i* and *j* is a communicative neuron, the benefit they each will receive is *β*(Δ*ϕ*_*ij*_) = *β*(0)[1 + cos (*ϕ*_*i*_ − *ϕ*_*j*_)]/2. If neither is communicative, they each will get zero benefit. (*b*) The 2 × 2 payoff matrix from the perspective of the row individual, arising from the scenario where the two neurons possibly take opposite communicative strategies. Depending on the payoff parameters *B*(0) and *β*(Δ*ϕ*), the payoff matrix given in (*b*) can encompass a wide range of game types, from snowdrift games to the Prisoner’s Dilemma to coordination games to mutualism, as characterized in (*c*). (Online version in colour.)

Depending on the choice of the model components *B*, *β* and *c*, the resulting game can take many forms. Figure 1*c* shows four regions of the parameter space linked to four classical games—coordination, mutualism, Prisoner’s Dilemma and the snowdrift games. The richness of these structures presents myriad possibilities during the evolution of synchronization and communication as the analysis of the evolutionary dynamics of these classical games can have very different outcomes.

We approach the analysis using the two fundamental parameters in the EGT framework—the mutation rate (*μ*) and the selection strength (*δ*)—as guideposts. There are two standard simplifying assumptions in the EGT literature: weak selection (when *δ* ≪ 1/*n*) [18,22] and low mutation (when *μ* ≪ 1/*n*) [20,23]. We explore the conditions under which communicative strategies are favoured under various combinations of these two standard assumptions. Analytically, we have techniques for several different regimes: weak selection and low mutation (*δ* ≪ 1/*n*, *μ* ≪ 1/*n*), strong selection and low mutation (*δ* ≫ 0, *μ* ≪ 1/*n*), low mutation but any selection strength (*μ* ≪ 1/*n*), and weak selection but any mutation rate (*δ* ≪ 1/*n*). Each of these, in turn, is addressed in the Results section below. Current analytic techniques cannot effectively approach cases when both the mutation rate and selection strength are large [20–24], and thus we reserve such explorations for future work.

When the mutation rate is very small, we can assume that after a mutation, the system will reach an equilibrium before the next mutation occurs and study a pairwise invasion model in the first two regimes. We construct a population where all but one oscillator has the same strategy with the idea that the different strategy arose due to a mutation. This allows us to explore two relevant questions about robustness and fragility of the system. For example, is a communicative equilibrium robust to perturbation, i.e. if a mutation introduces non-communication into an otherwise communicative population, does it persist? Similarly, does introduction of communication into a non-communicative equilibrium necessarily lead to a completely communicative new equilibrium?

The simplification to two strategies and, in particular, the restriction to only two phases mitigates one of the limitations of our framework. Since there are only two phases, the role of payoffs based on the difference in phase is more limited than if there were many options for phase values. A consequence is that the phase and communication trait evolve in tandem, *de facto* combining these traits into a single feature.

For the second pair of regimes, where we make either only the low mutation assumption or only the weak selection assumption, we explore the more general case where oscillators can adopt any of *d* distinct phases. Results from these analyses are applicable to a wider array of questions as they apply to systems with heterogeneous initial strategy distributions, allowing oscillators to take on more possible phases, introduces the possibility of more exotic outcomes like those we see in some Kuramoto systems such as phase-locking and cluster synchronization. Most obviously, this set-up moves beyond the limitation of having only two phases but re-introduces the unified timescale of trait coevolution discussed above. A gain in this generalization is the ability to understand the impact, if any, of the varied payoffs between the different strategies, as adding a full complement of phases allows impact on the trait evolution from the entire payoff matrix.

In what follows, we present results from several existing analytical frameworks in the literature as well as from simulation of the system. While there are variations in the results, a basic and fundamental rule is revealed throughout—if the benefit of synchronization exceeds twice the cost of communication, the system will evolve to complete communication and synchronization.

## 3. Results

To analyse our model, we use both analytic techniques and simulation. To derive analytical insights, we consider two types of scenarios: (1) the general case of evolutionary dynamics of multiple 2*d* types of neurons for any selection and low mutation, as well as for weak selection and any mutation, and followed by (2) simpler cases concerning pairwise invasion dynamics for both weak selection and strong selection limits. The latter scenario helps provide intuitions for understanding our general theoretical results involving multiple 2*d* strategy types under low mutation limit. In each scenario, we are able to apply existing evolutionary game theoretic techniques to explore conditions under which communicative strategies are favoured. In particular, in the limit of low mutation (as detailed in §5 of the electronic supplementary material), we prove that the condition for natural selection to favour communicative strategies, that is, for the long-term frequency of all communicative strategies (*C*, *) to be greater than one-half, is

3.1 $$B(0)>\frac{2c(n-1)}{n-2}.$$

This surprisingly simple condition is derived using the embedded Markov chain approach under the low mutation limit [23,24] and holds for a wide range of model parameters such as for any selection strength (*δ* > 0) and for arbitrarily many types (2*d* ≥ 2).

To provide helpful intuition for this condition with finite size correction (3.1), we consider the simpler scenario concerning pairwise competition since the low mutation limit implies that the fate of a new mutant (either reaching fixation or becoming extinct) is determined before the next mutant arises in the population. In other words, there are at most two types present at any time of the evolutionary process under the low mutation limit.

Accordingly, in the pairwise invasion scenario, we first assume that selection is weak, *δ* ≪ 1/*n*, and mutation is low, *μ* ≪ 1/*n*, to show how communicative strategies benefit from selective pressure. To make this precise, we compute the *fixation probability* of strategy *E* = (*C*, *ϕ*_*j*_), denoted *ρ*_*E*_, which is defined as the probability that, in a population with one strategy-*E* individual and *n* − 1 individuals with strategy *F* = (*N*, *ϕ*_*k*_), the process is absorbed into the state where all individuals have strategy *E* [13]. We similarly define *ρ*_*F*_ as the probability that, in a population with one strategy-*F* individual and the rest strategy *E*, the process is absorbed into the state with uniform strategy *F*. In §3 of the electronic supplementary material, we show that *ρ*_*E*_ > 1/*n* when

3.2 (n−2)B(0)+(n−2)β(Δϕ)>3c(n−1).

Therefore, if *n* ≫ 1, we get the following simpler condition that specifies exactly when strategy *E* has greater evolutionary success than a strategy under the neutral process

3.3 $$\rho_{E}>\frac{1}{n}⟺B(0)+\beta (\Delta \phi )>3c.$$

We note that this condition concerning the probabilistic invasion success of a single mutant *E* under the weak selection limit can be envisioned as if the interior unstable equilibrium of a coordination game is less than 1/3 in the corresponding deterministic replicator dynamics [18].

In examining when communicative strategies are favoured over non-communicative ones in pairwise invasion dynamics (i.e. *ρ*_*E*_ > *ρ*_*F*_—see electronic supplementary material, §3 for more details), we find that the ratio of *ρ*_*F*_ to *ρ*_*E*_ is

3.4 $$\frac{\rho_{F}}{\rho_{E}}=e^{\delta ((n-1)c-(\left(n-2\right)/2)B(0))}.$$

And so *ρ*_*E*_ > *ρ*_*F*_ if and only if

3.5 $$(n-1)c<\frac{n-2}{2}B(0).$$

Again, for sufficiently large populations (*n* ≫ 1), we get the simpler condition

3.6 $$\rho_{E}>\rho_{F}\;⟺\;B(0)>2c.$$

Aside from finite population correction, the condition (3.6) (also known as the risk dominance condition [18]) can also similarly be envisioned in deterministic replicator dynamics where the unstable interior equilibrium is less than 1/2 (the expected payoff of a communicative neuron is greater than that of a non-communicative one when both types are equally abundant in the population) [25].

Turning to the strong selection limit, in electronic supplementary material, §4.1, we consider strong selection (*δ* ≫ 1) and low mutation (*μ* ≪ 1). When there are few communicative strategy individuals in the population, communication is favoured when

3.7 $$\beta (\Delta \phi )>\frac{c(n-1)}{n-2}.$$

In a sufficiently large population, equation (3.7) implies that communication is favoured when

**Figure 2.**
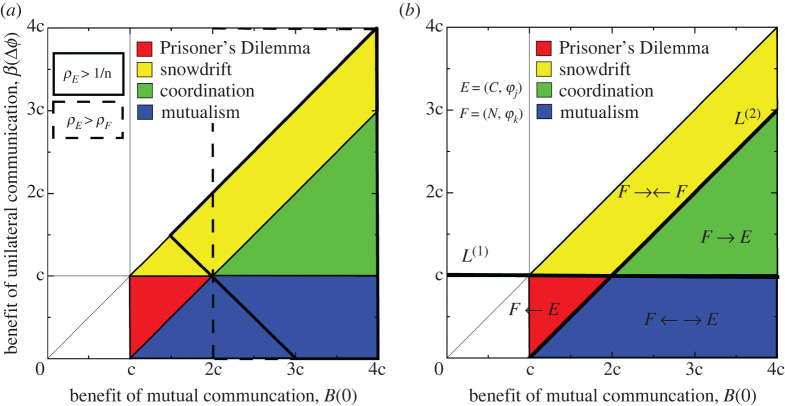
Pairwise invasion dynamics provide intuition for understanding evolutionary dynamics of multiple strategies under the low mutation limit. (*a*) Classical game regimes for different values of *B*(0) and *β*(Δ*ϕ*) under the assumption of weak selection and large population limits. Also marked are regions where *E* = (*C*, *ϕ*_*j*_) has a selective advantage, either over a neutral strategy (heavy line) or over the strategy *F* = (*N*, *ϕ*_*k*_) (dotted line). (*b*) Game regimes for different values of *B*(0) and *β*(Δ*ϕ*) in the case of strong selection. Above lines *L*^(1)^ and *L*^(2)^, strategy *F* is favoured when there are more strategy-*E* individuals, and strategy *E* is favoured when there are more strategy-*F* individuals. Below *L*^(2)^ and above *L*^(1)^, strategy *E* is always favoured, while strategy *F* is always favoured in the region below *L*^(1)^ and above *L*^(2)^. Finally, below both lines, strategy *E* is favoured when there are more strategy-*E* individuals, and strategy *F* is favoured when there are more strategy-*F* individuals in the population. (Online version in colour.)

3.8 β(Δϕ)>c,

which is depicted in figure 2*b* as the region above line *L*^(1)^. When there are few non-communicative strategy individuals in the population, communication is favoured when

3.9 $$\beta (\Delta \phi )<B(0)-\frac{c(n-1)}{n-2}.$$

Again, when the population is sufficiently large, this implies that communication is favoured if and only if

3.10 β(Δϕ)<B(0)−c,

which is represented by the region below line *L*^(2)^ in figure 2*b*. Together, despite having been derived for finite populations under strong selection, conditions (3.8) and (3.10), which reflect the large population limit, coincide with the classical ESS condition for determining invasion success of initially rare mutants. More interestingly, they also divide the parameter space into regions where the game dynamics differ, as seen in figure 2 (cf. figure 1*c*). Condition (3.8) corresponds to the region above line *L*^(1)^, while condition (3.10) corresponds to the region to the right of line *L*^(2)^. The strong limit results here can give us helpful intuition about the conditions for natural selection to favour communicative strategies over non-communicative ones. In the region *F* → ←*E* (characterized by snowdrift games), a single mutant, either *E* or *F*, can invade, but cannot fixate in any finite time, as the dynamics are ‘trapped’ around the interior coexistence equilibrium of snowdrift games, which is stable in infinite, well-mixed populations [24]. In the region *F* ← *E* (where neither condition (3.8) or condition (3.10) are satisfied, characterized by Prisoner’s Dilemma), communicative strategies *E* cannot invade at all and the system is dominated by non-communicative ones. In the region *F* → *E* (characterized by mutualism), communicative strategies *E* always can invade and take over resident non-communicative population. In the region *F* ← →*E* (characterized by coordination games), neither *E* nor *F* can be successfully invaded by initially rare mutants as the systems tend back towards their initial states. Taken together, we have fully characterized the possible game types of pairwise interactions in neuronal populations and in particular their implications for strategy selection conditions in the strong selection limit.

In the presence of 2*d* strategies and under the assumption of low mutation (*μ* ≪ 1/*n*), we find that for a sufficiently large number of oscillators communicative strategies (C,⋆) are favoured by the selection process if and only if *B*(0) > 2*c*. More specifically, we show that the frequency of (C,⋆) strategies is higher than that of the (N,⋆) strategies at stationarity if and only if *B*(0) > 2*c*. For 2*d* strategies under the assumption of weak selection (*δ* ≪ 1/*n*) and any mutation rate, we find that

3.11 $$\frac{1}{2}(B(0)-2c)+\mu \frac{n}{4}(\bar{B}-2c)>0,$$

where $\bar{B}$ is an average of the function *B* over all differences of phases: $\bar{B}=(1/d)(B(0)+B(d/2)+2\sum_{q=1}^{\lfloor d/2\rfloor -1}B(q))$. We note that in the limit of rare mutations, *μ* ≪ 1/*n*, the condition becomes *B*(0) > 2*c*. Hence, this condition, derived using the approach of Antal *et al.* in [22] in the limit of weak selection, also confirms the previous more general result that *B*(0) > 2*c* holds for any selection strength when mutations are rare. More details on these last cases can be found in electronic supplementary material, §3 and 4.

**Figure 3.**
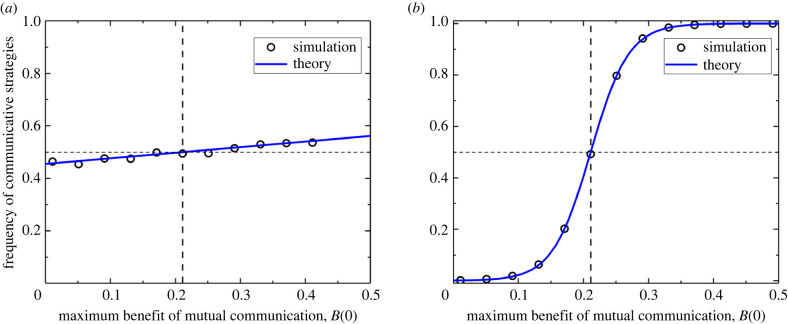
Excellent agreement between simulation results and analytical predictions. The plots (*a*,*b*) show the long-term frequency of communicative strategies (solid lines: theoretical predictions; empty circles: simulation results) as a function of the maximum benefit of mutual communication *B*(0). As given in (*a*,*b*), our predictions show that in the limit of rare mutations (*μ* ≪ 1/*n*), the long-term frequency of communicative strategies is favoured by natural selection (that is, the frequency of (C,⋆) strategies is greater than 1/2—vertical dashed line in (*a*,*b*)) if *B*(0) > 2*c*(*n* − 1)/(*n* − 2). Model parameters: population size *n* = 20, number of discrete phases *d* = 20, selection strength (*a*) *δ* = 0.005 and (*b*) *δ* = 0.2, cost of communication *c* = 0.1, maximum benefit of unilateral communication *β*(0) = 0.95*B*(0), and mutation rate *μ* = 0.0001. The simulation results are averaged over 2 × 10^9^ time steps. (Online version in colour.)

To further examine the more complex cases where there are more than two strategies present, we simulate the Moran process associated with our set-up in a finite, well-mixed population of size *n* to both validate and extend our analytical results. Our basic set-up begins with *n* = 20 oscillators and *d* = 20 discrete phases. We set the cost of communication at *c* = 0.1, a low mutation rate of *μ* = 0.0001, and the maximum benefit of unilateral communication at *β*(0) = 0.95 *B*(0) as we vary maximum benefit of bilateral communication, *B*(0), and the selection strength, *δ*. For a weak selection strength of *δ* = 0.005, our theoretical results predict that we should see communicative strategies favoured if *B*(0) > 2*c*(*n* − 1)/(*n* − 2) ≈ 0.21. Simulation results averaged over 2 × 10^9^ time steps, as shown in figure 3*a*, match closely with the prediction. As seen in that figure, the prediction is roughly linear, crossing the 50% threshold marking the point at which the population has a majority of communicating oscillators at the stationary distribution of the Moran process at exactly *B*(0) > 2*c*(*n* − 1)/(*n* − 2). The circles, denoting the averaged simulation results, track the line closely and also cross the 50% threshold at just about the same point. Looking to the strong selection regime, repeating our simulations after changing the selection strength to *δ* = 0.2 shows similarly excellent agreement between predicted and simulated results, as seen in figure 3*b*. There, the predicted results form a sigmoid-like curve which crosses the 50% threshold at the same value of *B*(0). Circles, again representing the average over simulations, track the curve closely and cross the threshold near the same value of *B*(0). Taken together, this supports the existence of a transition point at *B*(0) = 2*c*(*n* − 1)/(*n* − 2) after which the systems favour communicative strategies. For systems where there are many different strategies, different sub-populations may be playing different games than one another due to the varied possibilities shown in figure 1. As strategies evolve, the system may move through these different games, each of which has a different impact on favouring communication. To explore these potentially rich dynamics, we present two experiments.

**Figure 4.**
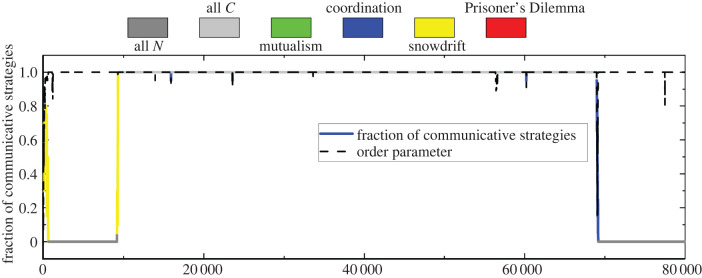
Coevolutionary dynamics of communicative strategies and phases in a finite, well-mixed population of neurons. The plot shows the time evolution of the order parameter (dashed line) which quantifies the degree of synchronization of neurons and the fraction of communicative neurons (solid line). The colour of the solid line indicates the most common type of game interactions in the population at each time step. We use the Moran process for the evolutionary update in our individual-based simulations. Model parameters: population size *n* = 20, number of discrete phases *d* = 20, selection strength *δ* = 0.2, cost of communication *c* = 0.1, maximum benefits *B*(0) = 1.5*c* and *β*(0) = 0.95*B*(0), and mutation rate *μ* = 0.0001. (Online version in colour.)

In the first experiment, we pick a configuration of parameters (*B*(0), *β*(0), *c*) = (0.15, 0.1425, 0.1) that place game play either in the snowdrift, Prisoner’s Dilemma, or mutualism regimes. Further, since 0.15 = *B*(0) < 2*c*(*n* − 1)/(*n* − 2) ≈ 0.21 we do not necessarily expect the evolution to favour communication. Figure 4 shows the results of this simulation experiment. We use the order parameter, *r*, to measure the extent of synchronization, which is defined as

$$r=\frac{1}{n}\left|\sum_{i=1}^{n}\;e^{i\phi_{i}}\right|.$$

The dotted line gives the value of the order parameter across the time steps. It begins at about 0.4 as the initial strategies are drawn at random. Over a small number of times steps (approx. 2000) it quickly moves very close to one, indicating phase synchronization, and stays there with only small perturbations for the rest of the simulation except at just before 70 000 time steps where it drops sharply before returning to values close to one. The structure of the population and the types of games its members are playing is not nearly as stable. The solid curve in the figure shows the most common type of game that oscillators are playing based on the categorization in figure 1 by colour of the line—between the coordination (blue), mutualism (green), Prisoner’s Dilemma (red), and snowdrift (yellow) games. In figure S1 in the electronic supplementary material, we plot the histogram of the types of aforementioned games that have been played. We also denote, in two shades of grey, if the population is completely non-communicative (dark grey) or completely communicative (light grey). Moving through the time steps, the most common game starts in the snowdrift region but quickly moves to the all non-communicative state despite the fact that simultaneously the order parameter jumps to almost one. After approximately 10 000 time steps, the game changes again to snowdrift briefly until all oscillators are communicative. This regime lasts the longest, until almost 70 000 time steps, when the game type becomes coordination briefly until the oscillators are again all non-communicative. The two big transitions come, at roughly 10 000 and 70 000 time steps, and represent changes similar to the invasion scenario—the introduction of a communicating oscillator by mutation into an otherwise completely non-communicative population at the point of the first transition, and the opposite situation in the second.

The results in the case of strong selection and low mutation inform our second experiment. Conditions (3.8) and (3.10) demonstrate both that *β*(Δ*ϕ*) must exceed the cost of communication, but also that *B*(0) must sufficiently exceed *β*(Δ*ϕ*). The second condition creates a new type of outcome in the portion of figure 1 corresponding to the snowdrift game. Rather than fixating on one of the two strategies, in this region the system comes into something close to a stable state in which it oscillates slightly around some mixture of communicative and non-communicative strategies unless *β*(Δ*ϕ*) dips below *c* or rises too close to *B*(0). In their analysis of the evolution of a population playing a snowdrift game [19], Antal & Scheuring demonstrate that while the system will eventually reach a steady state, the time it takes to do so is exponential due to the existence of a (unique) mixed evolutionarily stable strategy. In our setting, this poses the existence of systems that exhibit mixtures of communicative and non-communicative strategies that persist over many generations. To explore this, we use a choice of parameters, (*B*(0), *β*(0), *c*) = (0.25, 0.2375, 0.1), that place the game within the snowdrift regime of figure 1 but also in the region where we expect communication to be favoured in the long run.

**Figure 5.**
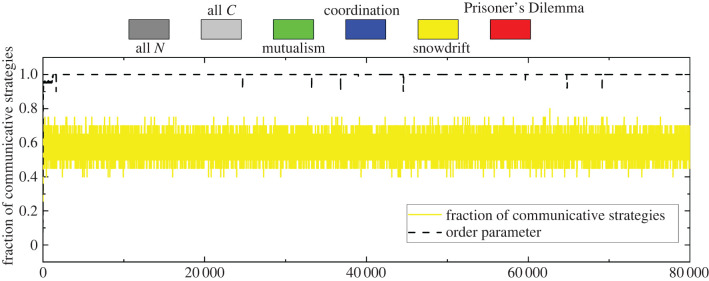
Snowdrift interactions provide a plausible mechanisms for the long-term coexistence of communicative strategies and their synchronization. The plot is similar to figure 4 except for strong selection (*δ* = 5). Once the population system wanders into the region of snowdrift games, it will be trapped in this state of coexistence for an exceedingly long time since the fixation time of snowdrift games is exponential [19]. In this scenario, synchronization is favoured despite the mixture of communicative and non-communicative neurons. Model parameters: population size *n* = 20, number of discrete phases *d* = 20, selection strength *δ* = 5, cost of communication *c* = 0.1, maximum benefits *B*(0) = 2.5*c* and *β*(0) = 0.95*B*(0), and mutation rate *μ* = 0.0001. (Online version in colour.)

Figure 5 shows the results of this simulation experiment. The structure of this figure is similar to figure 4, where the dotted line gives the order parameter and the solid line identifies the most common strategy by its colour and the fraction of communicative strategies by its height. The parameters of the simulation are identical to the previous experiment except the selection strength is very high, *δ* = 5, and *B*(0) = 2.5*c*. Like the previous simulation, the order parameter quickly moves to values close to one and remains there throughout the simulation with only small perturbations. In contrast to this stability, the fraction of communicative strategies bounces around between about 0.45 and 0.7 and the most common game played is the snowdrift game (see figure S2 in the electronic supplementary material).

## 4. Discussion

Our results describe various conditions under which synchronization in our model of the SCN is favoured. Of course, not all organisms exhibit behaviour that follows a circadian rhythm. For example, some organisms that live in extreme environments (the absence of light, for example), are going through extreme life stages (e.g. migration, reproduction), or are highly social fail to exhibit circadian behaviour [2,26]. It is also the case that not all organisms with circadian rhythms have a circadian system controlled by a ‘master clock’ like the mammalian SCN. For example, many fish are believed to have a more complex circadian clock arrangement involving a network of interconnected circadian units [2]. Our results, then, characterize when the SCN is able to function as the ‘master clock’ to maintain an organism’s circadian system.

So how do we interpret these results in terms of the biology? Given the framing of the model in §2, the pairwise invasion results when we have low mutation and weak selection tell us two things. First, so long as our basic condition, *B*(0) > 2*c*, holds, the system evolves towards synchronization and complete communication. Second, so long as *B*(0) > 2*c* and *B*(0) + *β*(Δ*ϕ*) > 3*c*, such systems are robust in the sense that invasion by a communicative oscillator is more likely to result in total communication than invasion by a non-communicator is to result in complete non-communication. Our results imply that organisms that see this level of benefits should develop a highly connected and interactive system of oscillators that facilitate synchronization. The second condition points to an avenue of fragility in the system—if *β*(Δ*ϕ*) < *c*, which could happen if *β* decays quickly and Δ*ϕ* is relatively large, then invasion by non-communication has a higher probability of becoming the stable state than a similar invasion by a communicator. Even under the assumption that the functions *B* and *β* are identical, this presents a stronger possibility of losing complete communication if a non-communicative oscillator is introduced via mutation with a phase sufficiently distant from the synchronous phase of the communicative portion of the system. This seems biologically implausible, as we would not expect a mutation that introduced non-communication to a stable synchronous system to lead to dismantling that system so long as the benefit is sufficiently high. Consequently, we interpret this as a constraint on the function *β*, providing a lower bound of *c* for its values. This condition appears explicitly when we consider strong selection and weak mutation. There (see (3.8) and (3.10)), we see that if *B*(0) − *c* > *β*(Δ*ϕ*) > *c*, then communication is favoured in either invasion set-up.

The intensity of the selection parameter *δ* in our model is an important, biologically plausible parameter that determines the stochasticity of the system. For weak selection, the system is close to neutral evolution and has a strong random drift effect, whereas for strong selection, the system is driven by a selection gradient (survival of the fittest), becoming essentially deterministic. Additionally, the mutation rate *μ* determines how the system travels across the simplex of the state space: for low mutation rate, the system stays on the edge of the simplex most of the time; in contrast, for high mutations, the system is close to the centre of the simplex where each strategy is equally present. While the specific *δ* and *μ* values may vary for real organisms [27], our theoretical prediction that communicative strategies are more abundant than non-communicative ones in the long run equilibrium if *B*(0) > 2*c* holds for any selection strength and low mutation limit. Moreover, we expect that increasing the mutation rate is likely to make communicative strategies harder to evolve [28].

The resulting mixture of communicative and non-communicative strategies that arise in the snowdrift region under strong selection presents an additional topological type of communication patterns for these systems. While fixation to (N,⋆) represents a topology with no communications and fixation to (C,⋆) yields all-to-all communications, this new case presents a mixture of connectivity of their communications—we would expect about two-thirds of the oscillators to communicate to all others while the remaining one-third has no outgoing communications to any other oscillators. Despite the simplified assumptions in our model, we feel this case most faithfully reflects what is known about the connectivity in the mammalian SCN. There, neurons often have connections to nearby neurons and, much less often, connections to distant ones. Moreover, different areas of the SCN have different connectivity patterns with more connections in some areas and less in others. While our model is too crude at this stage to mimic these subtleties, the existence of a mixed topological type of communication pattern lends support to the idea that generalizations of the model can produce similar heterogeneity to the biological network.

Turning to the more general cases with more than two phases, the results of evolutionary dynamics of multiple strategies for the low mutation limit and any selection strength as well as for weak selection and any mutation rate add further texture and support to the pairwise invasion results. For low mutation regimes, our analysis for any selection strength demonstrates that the basic cost–benefit condition, *B*(0) > 2*c*, persists even in a much more heterogeneous environment of strategies. These mixed environments are more biologically plausible, given the diversity of possible phases, and the reiteration of this condition reinforces its ubiquity. Our analysis of the weak selection limit for the case of multiple strategies, on the other hand, provides a window into the otherwise unexamined case of higher mutations. For low selection strength, the conditions encapsulated in equation (3.11) show that in conditions with high mutation rates, the more stringent requirement of $\bar{B}>2c$ is required for a strategy to be successful. This supports our expectation that higher mutation rates make communicative strategies harder to evolve.

In addition to the original Moran (birth–death) process considered in this work, its dual death–birth process, among others, is also often used to model evolutionary dynamics [29]. We confirm that these two update rules yield almost identical results for modelling neuronal interactions in well-mixed populations, which are equivalent to complete graphs [30]. However, they can lead to drastically different results for evolutionary dynamics in structured populations [29]. Prior work has pointed out that cooperation can never be favoured under the birth–death rule for evolutionary Prisoner’s Dilemma games on networks [29]. As neuronal interactions in the present model go beyond Prisoner’s Dilemma, it is of interest for future work to systematically compare how evolutionary outcomes are impacted by the specific update rules along with different network topologies [31].

## 5. Conclusion

We see two main contributions of our EGT framework for coupled oscillatory systems. First, we provide an alternative set-up for Kuramoto-type systems where it is easy to introduce evolutionary constraints on the connectivity of the system. This allows the study of the impact of these constraints and enables us to draw conclusions or conjectures about the structure of biological oscillatory systems. Topological properties of networks of coupled oscillators play a critical role in determining whether and how such a system will synchronize. Differences in topology can promote strong synchronization or weaker partial synchronization in a dizzying array of patterns—waves [32], chimeric states [33], pinwheels [33], cluster synchronization [34–38] and combinations of these. On the other hand, researchers in EGT have explored the impact of topology on the emergence of cooperation among agents in a structured population. Across these examples, we see a variety of outcomes—systems that converge to complete cooperation, complete defection, or mixed populations of defectors and cooperators—and a large body of work delineates topological structures that facilitate cooperation [29,39–42]. A recent sequence of papers describe topological statistics and signatures that push a system towards cooperation in pairwise interactions [43–45] and high-order interactions [46]. Our work provides a first step in exploring how these topologies arise in the context of evolutionary processes, providing a connection between these two lines of research in the special case of oscillatory systems. While this initial work applies only to a simple case allowing only binary connectivity—an oscillator is connected to all the oscillators or none of them—the framework easily adapts to more flexible modes of connectivity. Despite this simplification, our analysis reveals some of the complexity witnessed in the literature in identifying cost–benefit trade-offs that lead to fully connected, completely disconnected or partially connected networks.

Second, our set of initial results about this system reveal both baseline necessary conditions for synchronization as well as some more nuanced results in either high mutation or strong selection environments. Our analysis both quantifies and extends the intuitive conclusion that the benefits of synchronization must outweigh the costs substantially for an oscillatory system to develop connectivity in its support. In the more extreme evolutionary environments of high mutation or strong selection, the higher requirements for the benefit point to an additional evolutionary mechanism at play. As both selection strength and mutation rate can be different among different organisms as well as change over time in a single organism, periods of either condition place higher selection pressure on strategies with better fitness with respect to the benefit of synchronization while simultaneously producing barriers to achieving complete communicability and synchronization resulting in heterogeneous states that can persist for long periods of time. Moving into these heterogeneous states provides the system a chance to explore more areas of the parameter space and consequently provide the opportunity for new, and potentially more fruitful, configurations.

In sum, our work derives analytical evolutionary conditions for communication and synchronization behaviour of neurons to be favoured by natural selection in a variety of scenarios, supported and extended by simulation results. Sharing in common of these characterized evolutionary constraints is a surprisingly simple condition that the benefit of mutual communication and synchronization needs to be greater than *twice* the cost of doing so. Our theoretical and simulation results may help shed new insights into the widespread synchronization behaviour in populations of oscillators. While our work is motivated primarily by the mechanisms associated with circadian rhythms, we expect that this framework will be valuable in extending our understanding of other systems of coupled oscillators and the processes they model, such as synchronization in the flashing of fireflies or the chirping of crickets [47,48].

## Data Availability

All pertinent analysis has been included in the main text and in the electronic supplementary material [49].

## Endnote

1 Δ*ϕ*_*jk*_ = |*ϕ*_*j*_ − *ϕ*_*k*_| if |*ϕ*_*j*_ − *ϕ*_*k*_| ≤ *π*; otherwise Δ*ϕ*_*jk*_ = 2*π* − |*ϕ*_*j*_ − *ϕ*_*k*_| (as shown in figure 1*a*).
